# Outer Membrane Vesicles as a Candidate Vaccine against Edwardsiellosis

**DOI:** 10.1371/journal.pone.0017629

**Published:** 2011-03-08

**Authors:** Seong Bin Park, Ho Bin Jang, Seong Won Nho, In Seok Cha, Jun-ichi Hikima, Maki Ohtani, Takashi Aoki, Tae Sung Jung

**Affiliations:** Aquatic Biotechnology Center, College of Veterinary Medicine, Gyeongsang National University, Jinju, South Korea; Charité-University Medicine Berlin, Germany

## Abstract

Infection with *Edwardsiella tarda*, a Gram-negative bacterium, causes high morbidity and mortality in both marine and freshwater fish. Outer membrane vesicles (OMVs) released from Gram-negative bacteria are known to play important roles in bacterial pathogenesis and host immune responses, but no such roles for *E. tarda* OMVs have yet been described. In the present study, we investigated the proteomic composition of OMVs and the immunostimulatory effect of OMVs in a natural host, as well as the efficacy of OMVs when used as a vaccine against *E. tarda* infection. A total of 74 proteins, from diverse subcellular fractions, were identified in OMVs. These included a variety of important virulence factors, such as hemolysin, OmpA, porin, GAPDH, EseB, EseC, EseD, EvpC, EvpP, lipoprotein, flagellin, and fimbrial protein. When OMVs were administrated to olive flounder, significant induction of mRNAs encoding IL-1β, IL-6, TNFα, and IFNγ was observed, compared with the levels seen in fish injected with formalin-killed *E. tarda*. In a vaccine trial, olive flounder given OMVs were more effectively protected (p<0.0001) than were control fish. Investigation of OMVs may be useful not only for understanding the pathogenesis of *E. tarda* but also in development of an effective vaccine against edwardsiellosis.

## Introduction

Outer membrane vesicles (OMVs) are spherical blebs of average diameter 10–300 nm that are naturally released from Gram-negative bacteria into the environment [1]. Although the budding mechanisms are unclear, it has been shown that OMVs are continuously produced during growth of various Gram-negative bacteria including *Escherichia coli*, *Helicobacter pylori*, *Neisseria meningitidis*, *Pseudoaltermonas antarctica*, *Pseudomonas aeruginosa*, *Shigella flexneri*, and *Vibrio cholerae* [2]–[7]. Such vesicles are known to contain lipopolysaccharide (LPS), lipoproteins, outer membrane, periplasmic, and cytoplasmic proteins, DNA, and RNA [1], [8]–[10], and have been suggested to be involved in exclusion of competing bacteria, conveyance of proteins or genetic material to other bacteria, and presentation of virulence factors to the host [1].

*Edwardsiella tarda* is the causative agent of edwardsiellosis in a variety of cultured freshwater and marine fish, including channel catfish *Ictalurus punctatus*, olive flounder *Paralichthys olivaceus*, Japanese eel *Anguilla japonica*, red sea bream *Pagrus major*, mullet *Mugil cephalus*, and turbot *Scophthalmus maximus* [11]–[16]. Edwardsiellosis has been implicated in the mass mortality of olive flounder, which is the main mariculture species of South Korea. Typical clinical symptoms of *E. tarda* infection in olive flounder are exophthalmia, enlargement of the spleen, malodorous ascites, and rectal hernia [17].

A number of studies have shown that vaccination using outer membrane proteins results in development of protective effects against *E. tarda* infection [18]–[21]. In addition, the outer membrane proteins of *E. tarda* include several important virulence factors that play key roles in pathogenicity [17]. Virulence factors of *E. tarda* that have been investigated include dermatotoxin, hemolysins, catalase, outer membrane proteins, EseDs, and glyceraldehyde-3-phosphate dehydrogenase (GAPDH) [19], [22]–[24].

Previous proteomic studies indicated that both outer membrane proteins and LPS in OMVs might play roles as pathogen-associated molecular patterns (PAMPs) delivered to the host innate immune system, and could thus elicit immune responses [25], [26]. Because OMVs have antigenic properties, such vesicles have been investigated as useful candidate vaccines against Gram-negative bacterial infections [27]–[29]. For example, a *Neisseria meningitidis* serogroup B vaccine was successfully developed using derived OMVs; 55 million doses have been administered to date [30].

However, none of OMV protein composition, antigenicity, or vaccine efficacy has been studied in bacteria pathogenic for fish, especially the bacterium *E. tarda*. Thus, we investigated the possibility of using OMVs released from *E. tarda* as a vaccine against edwardsiellosis, based on both a proteomic study and cytokine induction assays.

## Results

### TEM examination of OMVs

**Figure 1:**
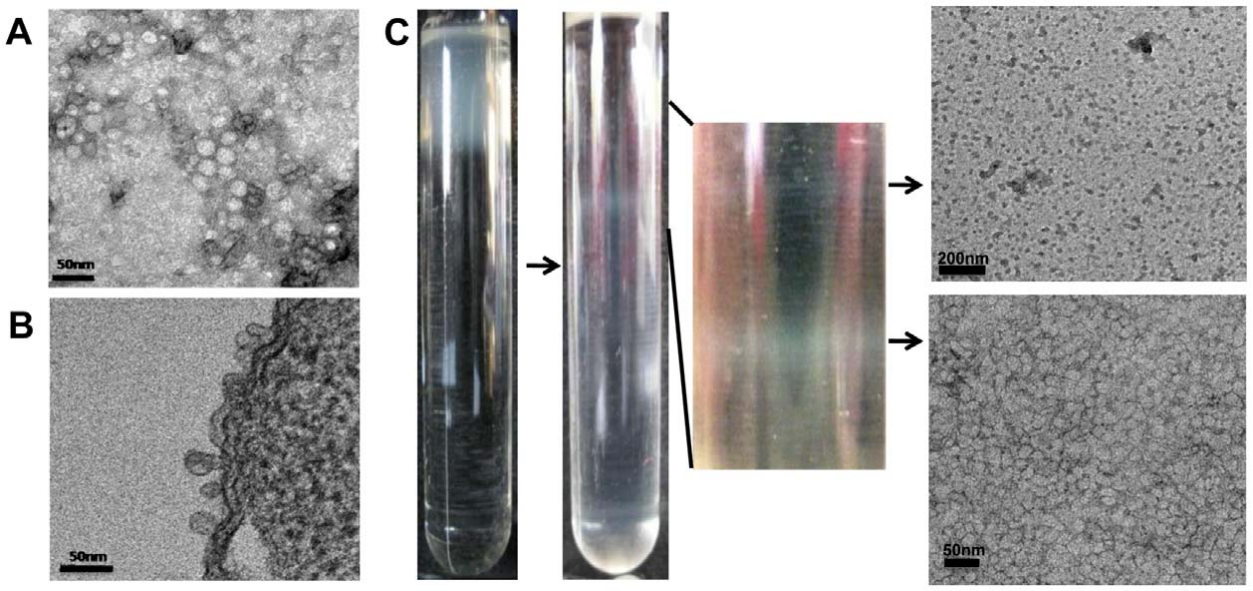
Transmission electron micrographs (TEMs) of outer membrane vesicles (OMVs) released from *Edwardsiella tarda* ED45. (A) Thin-section TEM of OMVs released from an ED45 cell. Bar = 50 nm. (B) Negatively stained TEM of concentrated OMVs of ED45. Bar = 50 nm. (C) OMVs purified on a sucrose density gradient. The lower fraction is composed of OMVs.

Numerous ovoid-to-round-shaped blebs were evident on the surface of ED45 cells when thin sections were stained to show OMVs (Figure 1-A). The supernatant concentrate contained OMVs that were round in shape, ranged from 10–40 nm in diameter, and contained electron-dense substances (Figure 1-B). Because cell debris and pili were observed upon negative staining, OMVs suspended in PBS were purified by discontinuous sucrose gradient centrifugation prior to protein analysis and *in vivo* immunogenicity testing. After centrifugation, two clear white bands were evident in the centrifugation tube, and the materials therein were examined by TEM (Figure 1-C). The upper band appeared to contain cell debris or aggregates, whereas the lower band was mainly OMVs, at a density of 1.185 g/ml. All further experiments were performed using these purified OMVs.

### Protein profiling of OMVs

**Figure 2:**
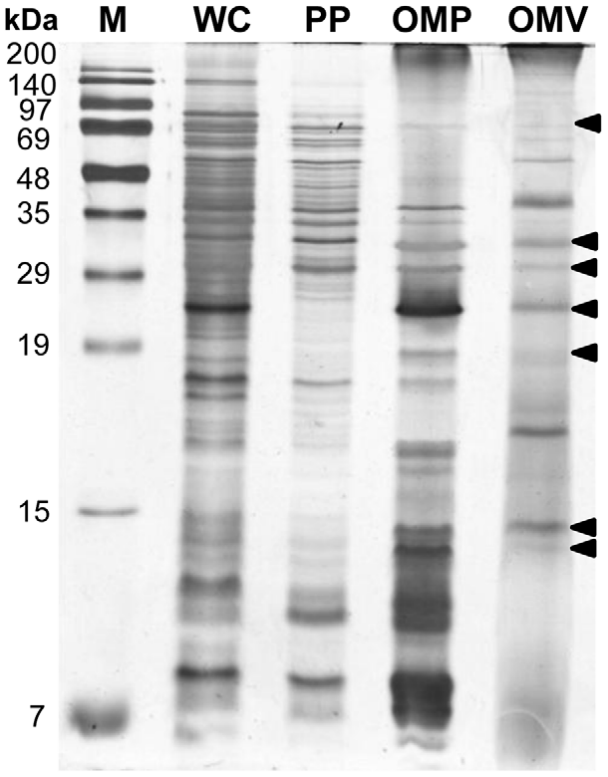
SDS-PAGE protein profiles of WCL, PPs, OMPs, and OMVs. Arrowheads indicate OMV polypeptides with molecular weights the same as those of OMPs. M: protein marker lane; WCL: whole cell lysate, PP: periplasmic proteins; OMPs: outer membrane proteins; OMVs: outer membrane vesicles.

**Figure 3:**
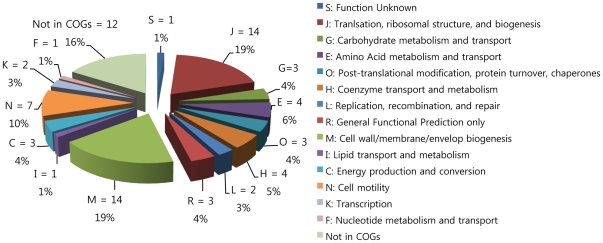
Functional classification of OMVs according to COG functional categories. The pie chart shows the numbers and percentages of identified proteins in each COG grouping. Individual protein assignment to COGs is shown in Table 1.

Purified OMVs were loaded onto 1D SDS-PAGE gels and proteins were visualized, after electrophoresis, using a silver staining method, and compared with proteins in WCL, PP, and OMP fractions. OMV proteins of molecular size 68, 31.5, 30, 24, 19, 14.5, and 14 kDa (Figure 2) were similar to those in the OMP fraction, but the 17-, 37-, and 54-kDa bands of OMVs were absent from the OMP sample. To identify the protein components of OMVs, the proteins were separated by 12.5% (w/v) SDS-PAGE and the gels were cut into 12 slices. Proteins in individual slices were analyzed by LC-ESI-MS/MS; acquired peptide mass data were analyzed using the MASCOT Daemon interface. A total of 74 proteins were identified in the *E. tarda* database (Table 1), and each slice contained at least 5 of these proteins (data not shown). Proteins were categorized into 15 different orthologous groups using the COG approach (Figure 3), indicating that the identified proteins were involved in both cellular processing and signaling (COG groups M, O, and N; 33% of proteins); information storage and processing (COG groups L, K, and J; 25% of proteins), and metabolism (COG groups I, H, G, F, E, and C; 21% of proteins). In total, 5% of proteins fell into poorly characterized categories (COG groups S and R), whereas 16% could not be identified in any COG grouping. The subcellular locations of the identified proteins were predicted using the PSORTb algorithm; this exercise suggested that 37 proteins were localized in the cytoplasmic space, 6 in the inner membrane, 16 in the outer membrane, and 9 in the extracellular space. The locations of six proteins could not be identified.

**Table 1:** Proteins of OMVs of *Edwardsiella tarda* identified by LC-ESI-MS/MS.

Accession number	Description	Mascot score	PI^a^	MW (Da)^b^	Protein matches	Functional category^c^	Subcellular localization^d^
gi|2244627	Hemolysin	288	5.99	165447	34	R	outer membrane
gi|291091829	Lipoprotein	131	9.67	33041	10	M	outer membrane
gi|267985457	long-chain fatty acid transport protein	429	6.45	47578	15	I	outer membrane
gi|267984860	murein lipoprotein	224	8.93	8384	9	M	outer membrane
gi|267985939	nucleoside-specific channel-forming protein Tsx	50	5.62	32786	2	M	outer membrane
gi|267984912	OmpA/MotB domain protein	44	9.62	17648	9	M	outer membrane
gi|25989456	outer membrane protein	575	5.27	47282	19	M	outer membrane
gi|267984281	outer membrane protein A	1482	7.66	38034	105	M	outer membrane
gi|253720368	outer membrane protein A precursor	1447	7.66	38048	104	M	outer membrane
gi|291089413	peptidoglycan-associated lipoprotein	52	5.93	18793	10	M	outer membrane
gi|267985578	peptidoglycan-associated outer membrane lipoprotein	89	5.92	18770	7	M	outer membrane
gi|267983927	putative hemolysin precursor	288	5.9	167296	29	R	outer membrane
gi|267983035	putative outer membrane lipoprotein	38	10.01	22250	2	M	outer membrane
gi|267984253	putative outer membrane porin F protein	303	5.03	40058	11	M	outer membrane
gi|73532672	putative virulence-related membrane protein	95	9.4	19858	5	-	outer membrane
gi|267985681	virulence-related outer membrane protein	910	8.89	20476	34	-	outer membrane
gi|291091795	lysophospholipid transporter LplT	36	9.08	34123	10	R	cytoplasmic membrane
gi|291089401	protein YdcF	36	5.28	28546	4	-	cytoplasmic membrane
gi|291089679	putative inner membrane protein	65	5.19	19700	2	S	cytoplasmic membrane
gi|291090200	transcriptional regulator, LysR family	33	6.46	35207	4	-	cytoplasmic membrane
gi|291089298	twin arginine-targeting protein translocase TatA	42	9.3	9330	21	K	cytoplasmic membrane
gi|267985130	ABC transporter-related protein	32	9.43	28704	33	N	cytoplasmic membrane
gi|267986212	30S ribosomal protein S13	146	10.58	13283	6	J	cytoplasmic
gi|267985854	30S ribosomal protein S16	63	8.36	6755	4	J	cytoplasmic
gi|267983753	30S ribosomal protein S2	37	6.61	26281	10	J	cytoplasmic
gi|267986226	30S ribosomal protein S3	44	10.27	25980	9	J	cytoplasmic
gi|267983384	30S ribosomal protein S6	51	5.29	15270	12	J	cytoplasmic
gi|267986235	30S ribosomal protein S7	120	10.3	17609	12	J	cytoplasmic
gi|267983534	30S ribosomal protein S9	96	10.94	14834	6	J	cytoplasmic
gi|267983533	50S ribosomal protein L13	33	9.91	16049	15	J	cytoplasmic
gi|291091309	6,7-dimethyl-8-ribityllumazine synthase	76	5.32	16195	5	H	cytoplasmic
gi|267983456	altronate hydrolase	31	5.78	54172	22	G	cytoplasmic
gi|291091008	asparagine–tRNA ligase	39	5.16	52655	7	J	cytoplasmic
gi|267984254	asparaginyl-tRNA synthetase	39	5.1	52605	7	J	cytoplasmic
gi|291092134	aspartate ammonia-lyase	50	5.32	53103	7	E	cytoplasmic
gi|291092137	chaperonin GroL	836	4.84	57456	65	O	cytoplasmic
gi|267985752	cobyrinic acid ac-diamide synthase	32	5.12	30638	6	H	cytoplasmic
gi|267983856	conserved hypothetical protein	36	10.34	13945	2	-	cytoplasmic
gi|22121758	Cpn60	54	4.46	19369	6	O	cytoplasmic
gi|267986210	DNA-directed RNA polymerase subunit alpha	68	5.03	36725	12	K	cytoplasmic
gi|55981975	EseD	166	5.34	21101	6	-	cytoplasmic
gi|291089872	formate acetyltransferase	35	5.71	85456	13	C	cytoplasmic
gi|267985188	formate acetyltransferase 1	35	5.65	85471	13	C	cytoplasmic
gi|222457929	glyceraldehyde-3-phosphate dehydrogenase	146	6.6	35684	11	G	cytoplasmic
gi|224382175	heat shock protein 60	54	4.41	17870	5	O	cytoplasmic
gi|267985045	hypothetical protein ETAE_2037	104	9.3	20384	10	-	cytoplasmic
gi|291092198	lysine decarboxylase	70	5.63	81254	15	E	cytoplasmic
gi|267983773	lysine decarboxylase 1	327	5.53	81313	33	E	cytoplasmic
gi|291091450	lysine decarboxylase, inducible	235	5.53	81368	34	E	cytoplasmic
gi|291089681	O-succinylbenzoate-CoA ligase	39	8.62	50218	7	IQ	cytoplasmic
gi|267985017	polypeptide-transport-associated domain protein	396	6.17	27889	24	-	cytoplasmic
gi|267983427	polyribonucleotide nucleotidyltransferase	130	5.2	76425	7	J	cytoplasmic
gi|267985748	putative integrase	32	9.73	44853	8	L	cytoplasmic
gi|267983991	riboflavin synthase beta-chain	76	5.65	16208	8	H	cytoplasmic
gi|267984414	ribose-phosphate pyrophosphokinase	143	5.25	34557	9	FE	cytoplasmic
gi|291088840	ribosomal protein S19	42	10.42	10380	6	J	cytoplasmic
gi|291091495	ribosomal protein S2	37	6.33	26830	13	J	cytoplasmic
gi|291089791	site-specific recombinase, phage integrase family	32	9.7	44682	9	L	cytoplasmic
gi|73532652	type III secretion system effector protein D	53	5.11	13695	4	N	cytoplasmic
gi|74474901	antigenic protein Et 46	55	5.2	43774	19	H	extracellular
gi|256599595	Chain A, Structure Of A Type Six Secretion System Protein	290	-	18043	16	-	extracellular
gi|61743025	EseC	597	6.15	53717	42	O	extracellular
gi|40287638	EvpC	308	5.71	18143	19	K	extracellular
gi|38016008	fimbrial protein	281	9.14	38109	12	-	extracellular
gi|117307392	flagella (r) hook associated protein HAP2	95	6.62	50267	33	C	extracellular
gi|24306148	Flagellin	55	4.88	43722	18	C	extracellular
gi|27808146	major fimbrial subunit protein	2000	7.71	18473	78	G	extracellular
gi|267983886	type III secretion system effector protein C	597	6.08	50911	37	O	extracellular
gi|61743023	EseB	287	5.51	21777	12	-	unknown
gi|237861343	EvpP	165	9.38	20991	11	E	unknown
gi|291092160	N-acetylmuramoyl-L-alanine amidase	47	10.81	60479	15	E	unknown
gi|267985878	outer membrane lipoprotein	217	9.56	33180	11	E	unknown
gi|291089780	pseudouridine synthase, RluA family	35	9.11	7339	4	IQ	unknown
gi|267983367	putative N-acetylmuramoyl-L-alanine amidase	47	10.56	59093	12	-	unknown

^*a*^Theoretical protein charge.

^*b*^Theoretical protein molecular mass.

^*c*^Functional categorization based on COGs. The abbreviations are shown in Figure 3. ‘-’ indicates ‘Not in COGs’.

^*d*^Subcellular localization was predicted using the PSORTb v3.0 algorithm.

### Immune responses in olive flounder

**Figure 4:**
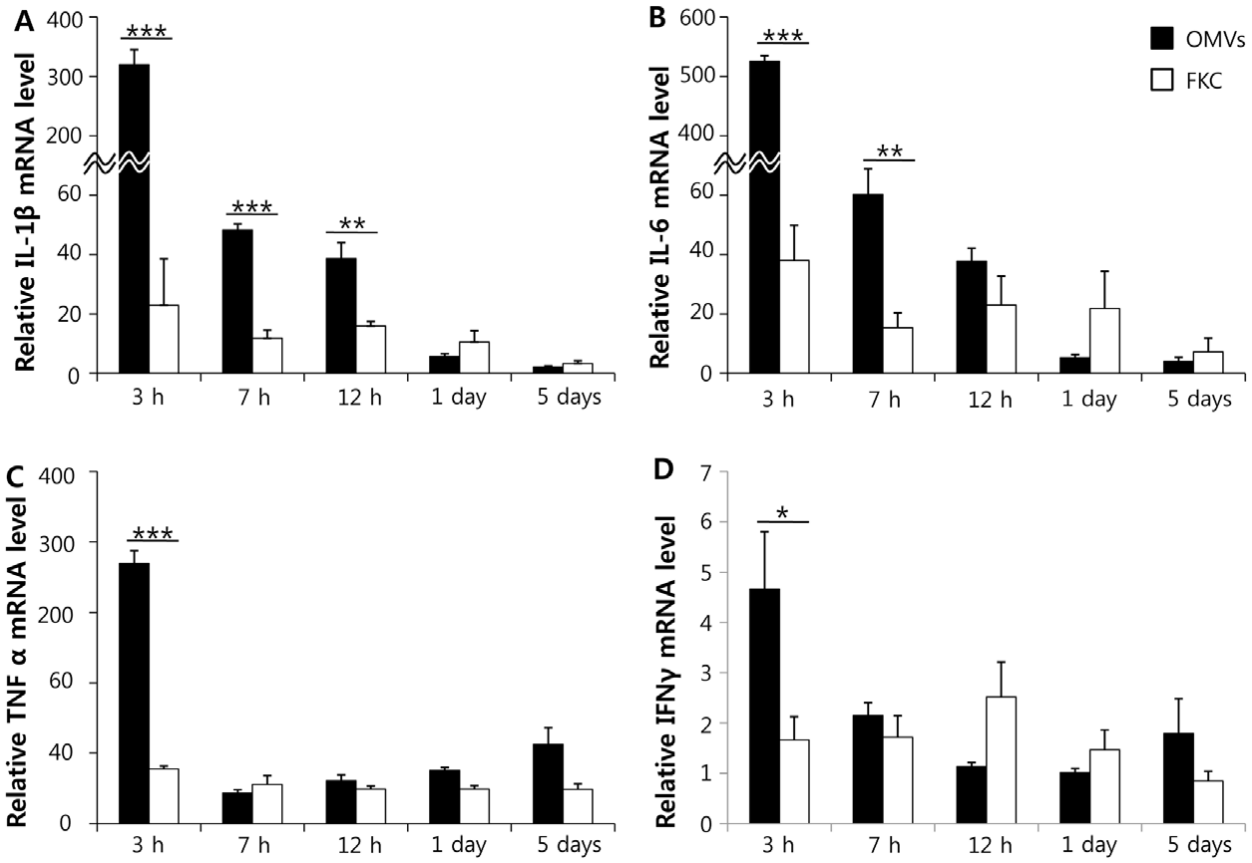
Relative induction of IL-1β (A), IL-6 (B), TNFα (C), and IFNγ (D) in olive flounder injected with OMVs or FKC, estimated using quantitative real-time PCR. OMV: group injected with outer membrane vesicles; FKC: group injected with formalin-killed ED45. *P<0.05, **P<0.01, and ***P<0.001. Bars indicate standard deviations. N = 4.

Based on the proteomic data, QRT-PCR was conducted to measure mRNA expression levels of IL-1β, IL-6, TNFα, and IFNγ in olive flounder injected with FKC or OMVs, to investigate whether OMVs could elicit host immune responses. After intraperitoneal injection of OMVs or FKC, kidneys of injected fish were sampled at 0, 3, 7, and 12 hpi; and 1 and 5 dpi (Figure 4). Fish injected with OMVs showed increased cytokine levels compared with those measured at 0 hours. IL-1β and IL-6 expression levels were induced 320- and 515-fold at 3 hpi, and these levels were maintained to 5 dpi. TNFα and IFNγ synthesis levels increased 4.8- and 7.6-fold at 3 hpi, compared with zero time measurements. Fish injected with FKC showed differences in IL-1β and IL-6 expression levels (compared with controls) at early time points, but neither the TNFα nor IFNγ synthesis level was distinct from that of 0-hour control values. When expression levels of IL-1β, IL-6, TNFα, and IFNγ in fish injected with OMVs were compared with those in fish injected with FKC, the cytokine levels of OMV-injected fish were significantly higher, at early time points, than in fish injected with FKC. The level of IL-1β expression in fish injected with OMVs differed significantly from that in fish injected with FKC, at 3, 7, and 12 hpi, whereas IL-6 levels were significantly different at 3 and 7 hpi. TNFα and IFNγ expression levels in fish injected with OMVs differed, at 3 hpi, from those in fish injected with FKC.

**Figure 5:**
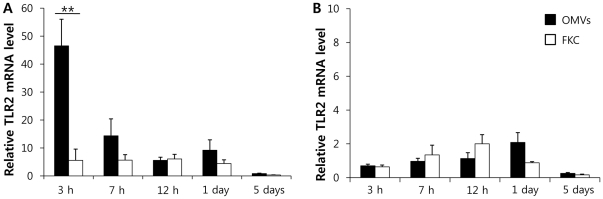
Relative induction of TLR22 (A) and TLR2 (B) in olive flounder injected with OMVs or FKC, estimated using quantitative real-time PCR. OMV: group injected with outer membrane vesicles; FKC: group injected with formalin-killed ED45. **P<0.01. Bars indicate standard deviations. N = 4.

mRNA TLR22 (toll-like receptor 22) and TLR2 expression levels, which are pattern recognition receptors (PRRs) in olive flounder, were measured to determine whether the innate immune response could be elicited by OMVs [31]. TLR22 levels in fish injected with OMVs were significantly higher than in fish injected with FKC. However, TLR2 levels did not differ (Figure 5).

### Protective effects of OMV injection into olive flounder

**Figure 6:**
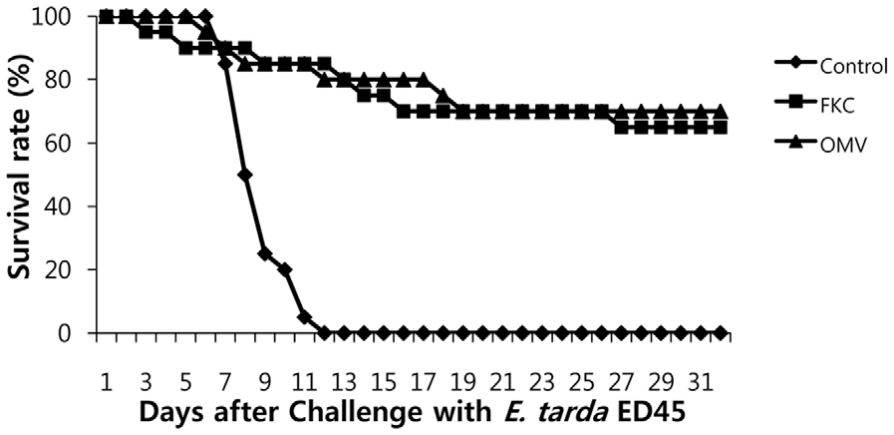
Survival rates of olive flounder challenged with ED45 four weeks after immunization. Control: PBS-injected group; FKC: group injected with formalin-killed ED45; OMV: group injected with outer membrane vesicles.

OMVs were administered to olive flounder as a candidate vaccine against *E. tarda* infection; control fish were injected with PBS (Figure 6). Fish injected with FKC were used as positive controls. After acclimatization of fish for 28 days, 1.1×10^4^ CFU/ml ED45 was used as an intraperitoneal challenge, and survival rates were recorded over 31 days. Control fish died from 6 dpi and all fish were dead at 11 dpi. OMV-vaccinated fish showed some mortality at 5 dpi; however, 70% of fish survived until 31 dpi. FKC-injected fish had a survival rate of 65%. OMV showed an RPS of 70%; this differed significantly from that of control (PBS) (p<0.0001).

## Discussion

Several Gram-negative bacteria produce OMVs [1]. In the present study, *E. tarda* was also shown to produce round OMVs, 10–40 nm in diameter, when grown in a liquid medium. Preparation of pure OMVs from bacterial supernatants is essential when studying the roles played by OMVs in both bacteria and their hosts [9], [10]. Recently, pure OMVs from bacterial supernatants have been obtained by filtration followed by density gradient ultracentrifugation [8], [25]. In the present study, homogenously sized *E. tarda* OMVs were separated from contaminants using such methods. Flagella, pili, and aggregated proteins were present in OMV samples before sucrose density gradient purification, but were removed during this step.

Two representative proteomic analysis methods have been suggested for the study of OMVs. Of these, the first is two-dimensional electrophoresis (2-DE) followed by matrix-associated laser desorption/ionization time-of-flight (MALDI-TOF) spectrometry, and the other is 1D SDS-PAGE followed by LC-ESI-MS/MS [10], [25], [26]. 2-DE is most commonly used to establish proteomic maps of bacteria, but limitations of the technique include poor separation of high-molecular weight and hydrophobic proteins [32]. However, 1D SDS-PAGE in combination with LC-ESI-MS/MS was effective in analysis of hydrophobic outer membrane proteins contained in OMVs [6]. In the present work, a total of 74 proteins were identified, 16 of which were characteristic in the outer membrane, as indicated by the PSORTb algorithm. However, a proteomic survey of the *E. tarda* outer membrane identified only 21 proteins by 2-DE analysis, and only 1 protein was confined to this membrane [33]. Therefore, 1D SDS-PAGE coupled with LC-ESI-MS/MS is more sensitive when used to analyze proteins of outer membranes or OMVs.

Some reports have suggested that OMV proteomes consist mainly of outer membrane and periplasmic proteins [34], but other proteomic studies found that OMVs contained proteins of various origin, including all of cytoplasmic, inner membrane, outer membrane, and periplasmic proteins [8], [25], [35]. By 1D SDS-PAGE, a 17-kDa band evident in OMVs could not be observed in outer membrane or periplasmic protein fractions. The PSORTb algorithm also indicated that proteins of OMVs originated not only from the outer membrane but also from cytoplasmic and extracellular compartments. These findings, together with those of previous proteomic studies, indicate that the OMV proteome includes proteins varying in subcellular origin, and other components.

LC-ESI-MS/MS analysis of OMVs showed that several proteins involved in pathogenesis, including hemolysin, outer membrane protein A (OmpA), GAPDH, EseB, EseC, EseD, EvpC, EvpP, lipoprotein, flagellin, and fimbrial protein, were present. Hemolysin is known to be required for cellular invasion and cytotoxicity [22]; hemolysin-negative *E. tarda* mutants could not invade human epithelial cell lines [36]. An ompA-negative *E. coli* strain invaded brain microvascular endothelial cells only with difficulty [37], [38]. GAPDH in the outer membrane is highly antigenic, and is considered to be a strong vaccine candidate to counter not only Gram-negative but also Gram-positive bacterial infections [19], [39]. EseB, EseC, EseD, EvpC, and EvpP are essential for *E. tarda* pathogenesis; these proteins are located on the cell surface and are responsible for pore formation [21], [40], [41]. Thus, OMVs released from *E. tarda* contain numerous virulence factors that may be important both for bacterial survival and to enhance immunogenicity in the natural host.

QRT-PCR demonstrated that the genes encoding all of IL-1β, IL-6, TNFα, and IFNγ were induced in fish injected with OMVs of *E. tarda*, compared with fish injected with FKC, especially at early post-injection time points. Similarly, mice injected with OMVs of *Bordetella pertussis* showed upregulation of mRNAs encoding IL-6 and TNFα, compared with the levels seen in animals injected with formalin-killed *B. pertussis* [27]. It is known that the primary host immune response is mediated by the proinflammatory cytokines IL-1β, IL-6, and TNFα [42]. Expression of such cytokines following injection of *E. tarda* OMVs indicates that the OMVs may initiate a proinflammatory cytokine cascade, including both recruitment and activation of macrophages and stimulation of an adaptive immune response. IFNγ, a Th1 cytokine, is also upregulated in fish injected with OMVs, and is known to enhance cell-mediated immunity and antigen presentation to macrophages [42]. Such observations may indicate that OMVs trigger an elevated host immune response, thus provoking host adaptive immunity, even though protein levels in OMVs are lower than those in FKC.

Innate immunity is an important first line of defense against invading pathogens, and recognition of PAMPs is achieved principally by PRRs [43]. In the present study, virulence factors, including lipoprotein, flagellin, and peptidoglycan, contained within OMVs of *E. tarda*, may have played roles as PAMPs, thus interacting with the innate immune system. In addition, OMVs are known to possess LPS and bacterial DNA that function as ligands for host PRRs [44], [45]. OMVs have been reported to be recognized by TLR2 and TLR9, both of which participate in IL-6 production *via* myeloid differentiation factor 88 (MyD88)-dependent pathways [45]. In the present study, TLR2 was not induced after injection with OMVs (compared with what was seen in positive control fish), whereas, in contrast, the TLR22 level was significantly increased. Therefore, the PRRs of olive flounder also participate in recognition of OMVs but the teleost detection mechanism for OMVs may differ from that of mammals.

The proteomic data, and the demonstrated immunostimulatory effects of OMVs from *E. tarda*, showed that OMVs exhibited high protective efficacy against *E. tarda* infection of olive flounder, with an RPS of 70% (p<0.0001). Other reports have described successful inhibition of *E. tarda* infection in laboratory experiments using various antigen preparations including formalin-killed and ghost cells, recombinant proteins, and outer membrane fractions [18]–[21]. However, these advances have not been translated into successful commercial vaccines. Although OMVs were no more effective as a vaccine than was FKC, vaccines of the latter type sometimes function poorly [46]. The OMV work of the present study offers a new insight into vaccine formulation. Thus, an acellular vaccine, or the use of acellular materials as a vaccine adjuvant, may be helpful in development of an effective vaccine protecting against edwardsiellosis in fish.

In conclusion, OMVs naturally released from *E. tarda* contained various important virulence factors originating from diverse subcellular locations. Such vesicles were able to induce synthesis of several proinflammatory cytokines, and may stimulate the host innate immune system, thus serving as PAMPs. To demonstrate vaccine efficacy, we injected olive flounder with OMVs, and found that such fish were protected to a significantly higher extent than were control fish. The present study on OMVs may lead to development of more effective vaccines, and may enhance our understanding of the roles of OMVs in hosts.

## Materials and Methods

### Bacterial strain and growth conditions

ED45, a virulent *E. tarda* strain isolated from the spleen of infected olive flounder, was used in the present study; the LD_50_ value was 1.2×10^3^ CFU/ml (data not shown). ED45 was cultured on Tryptone Soya Agar (TSA; Oxoid, Hampshire, England) or in Tryptone Soya Broth (TSB; Oxoid), supplemented with 2% (w/v) NaCl (termed the TSA-2 and TSB-2 media, respectively). ED45 was grown on TSA-2 at 25°C for 36 h; a single colony was inoculated into 25 ml TSB-2, and the culture grown with shaking to a cell density of 1×10^9^ CFU/ml. Next, 20 ml of this culture was added to 4 l TSB-2 and growth proceeded in a shaking incubator at 25°C for 15 h. After centrifugation at 5,000×*g* for 20 min, the supernatant was used for isolation of OMVs.

### OMV preparation

OMVs were collected and purified from the supernatant as described previously, with several modifications [25], [47]. The supernatant containing OMVs were filtered through a 0.45 µm pore-sized hollow fiber cartridge and concentrated using a hollow cartridge 100 kDa in size cutoff, employing a Quixstand benchtop system (GE Healthcare, Uppsala, Sweden). The concentrated supernatant was filtered through a 0.2 µm pore-sized vacuum filter to eliminate all remaining ED45 cells. Subsequently, OMVs were pelleted by ultracentrifugation at 150,000×*g* for 3 h at 4°C in an XL-90 ultracentrifuge running a 70Ti rotor (Beckman Coulter, Palo Alto, CA).

Pelleted OMVs were resuspended in phosphate-buffered saline (PBS; pH 7.2), layered onto a 30–60% (w/v) discontinuous sucrose gradient, and centrifuged at 200,000×*g* for 20 h at 4°C in a XL-90 ultracentrifuge running an SW41 Ti rotor, to obtain purified OMVs. Two clear bands were visualized, and the sucrose density in the vicinity of each band was measured by refractometry (Figure 1). After washing material from both bands (collected separately) with a 20-fold dilution of PBS, the materials were centrifuged at 150,000×*g* for 3 h at 4°C in a XL-90 ultracentrifuge. The final pellets were resuspended in PBS and protein concentrations were determined using a non-interfering assay (G-Biosciences, St. Louis, MO). The final preparations were stored at −80°C prior to use.

### Transmission electron microscopy (TEM)

ED45 cells were grown in TSB-2 at 25°C until an OD_600_ value of 1.0 was attained, centrifuged at 5,000×*g* for 30 min, and washed three times with PBS prior to preparation of ultrathin sections. After centrifugation at 5,000×*g* for 20 min, pellets were pre-fixed in 2% (v/v) paraformaldehyde for 3 h, washed, and fixed in 4% (w/v) osmium tetroxide for 2 h. After washing with PBS, the fixed pellets were dehydrated in a series of 70–100% (v/v) ethanol baths and embedded in epoxy resin. Sections were cut using a diamond blade (Diatome, Biel, Switzerland) fitted to an ULTRACUT UCT (Leica, Vienna, Austria), mounted on carbon-coated copper grids, and stained with 3% (w/v) uranyl acetate and lead citrate. To visualize OMVs after negative staining, samples were placed on 400- mesh carbon-coated grids for 2 min, washed with deionized sterile water (dd H_2_0), and negatively stained with 3% (w/v) uranyl acetate for 30 sec. All TEM images were acquired using a Technai 12 (FEI, Hillsboro, OR) operating at an acceleration voltage of 120 kV.

### Preparation of whole cell lysates, periplasmic proteins, and outer membrane proteins

Periplasmic proteins (PPs) and outer membrane proteins (OMPs) were purified as described previously [48]. Whole cell lysates (WCLs) of ED45 were obtained from cells grown in TSB-2 at 25°C, pelleted at 5,000×*g* for 30 min, and washed with PBS. Pelleted cell density was adjusted to 4 g/ml in 20% (w/v) sucrose dissolved in 20 mM Tris-HCl (pH 8.0), also with 0.1 M EDTA and lysozyme (600 µg/g cells). After incubation on ice for 40 min, 0.5 M MgCl_2_ (0.16 ml/g cells) was added and spheroplasts were removed by centrifugation (9,500×*g* for 20 min). The supernatant containing PPs was stored at −80°C until use. Spheroplasts were resuspended in ice-cold 10 mM Tris-HCl (pH 8.0) and sonicated for purification of OMPs. Following centrifugation at 8,000×*g* for 5 min to remove cells, the supernatant was centrifuged at 40,000×*g* for 1 h to pellet cell membrane material. Pellets were washed in 10 mM Tris-HCl (pH 8.0), resuspended in dd H_2_0, and freeze-thawed. The membranes were incubated in 0.5% (w/v) Sarkosyl (sodium N-lauroylsarcosinate; Sigma, St. Louis, MO) at 25°C for 20 min and OMP pellets were purified by centrifugation at 40,000×*g* for 1 h. Purified OMPs were resuspended in 10 mM Tris-HCl (pH 8.0) and stored at −80°C until use. Protein concentration was determined using a non-interfering protein assay (G-Biosciences, Hercules, CA).

### Electrophoresis and in-gel digestion

SDS-PAGE was performed using a 15% (w/v) acrylamide separating gel, according to the method of Laemmli [49]. Briefly, each resuspended WCL, PP, OMP, and OMV protein sample was mixed with 5×sample buffer (5∶1 v/w ratio of buffer to sample). The buffer contained 60 mM Tris-HCl, 25% (v/v) glycerol, 2% (w/v) SDS, 14.4 mM β-mercaptoethanol, and 0.1% (w/v) bromophenol blue. Samples were boiled for 10 min, cooled on ice, and centrifuged at 16,000×*g* for 20 min. Supernatants were subjected to SDS-PAGE analysis. Five microgram amounts of WCL, PP, OMP, and OMV proteins were loaded and proteins were detected by silver staining [50].

To achieve in-gel digestion, 20 µg amounts of OMVs were electrophoresed on a 12.5% (w/v) separating gel and stained with Bio-safe Coomassie G-250 (Bio-Rad, Hercules, CA). After cutting the gel into 12 slices, each slice was destained with a 500 µl amount of 40% (v/v) ethanol in 75 mM ammonium bicarbonate (ABC) and treated with DTT (0.0039 g dithiothreitol in 5 ml 25 mM ABC) and IAA (0.0509 g iodoacetamide in 5 ml 25mM ABC) solutions. Each gel slice was dehydrated using 300 µl acetonitrile (ACN) for 30 min at 37°C, dried, and used for in-gel digestion employing 20 ng/ml of sequencing-grade modified trypsin (Promega, Madison, WI) at 37°C overnight. Tryptic peptides were extracted into 30 µl 0.1% (v/v) formic acid and the solutions were sonicated for 10 min.

### LC-ESI-MS/MS and data analysis

LC-ESI-MS/MS (liquid chromatography electrospray ionization tandem mass spectrometry) analysis was carried out using a Thermo Finnigan Proteome X workstation with an LTQ linear ion trap and a MS apparatus equipped with NSI sources (Thermo Electron, San Jose, CA). Twelve microliter amounts of peptide mixtures were injected and loaded onto peptide trap cartridges (Agilent, Palo Alto, CA). Trapped peptides were eluted onto a 10 cm-long reverse-phase PicoFrit column packed in-house with 5 µm C18 resin of pore size 300 Å, and the peptides were next separated on an RP column using gradient elution. The mobile phase solutions were H_2_O and ACN, both containing 0.1% (v/v) formic acid, and delivered at a constant flow rate of 0.2 µl/min. The gradient commenced with 2.0% (v/v) ACN, rising linearly to 60% (v/v) ACN over 50 min, next increasing to 80% (v/v) ACN over the next 5 min, with a change to 100% H_2_O for the final 15 min. A data-dependent acquisition mode (m/z 300–1,800) was enabled, and each survey MS scan was followed by five MS/MS scans with the 30 sec dynamic exclusion option set. The spray voltage was 1.9 kV and the ion transfer tube temperature 195°C. The normalized collision energy was set to 35%.

The mzData file of tandem mass spectra was used to search the *E. tarda* database of NCBI downloaded on 9 July 2010, using the Mascot Deamon interface (Version 2.2.2, Matrix Science Inc., London, UK) supported by the Korea Basic Science Institute (KBSI; Yusung-gu, Daejeon, Korea). The peptide tolerance of the parent ion was adjusted to be 1 Da and the MS/MS tolerance was 0.8 Da. During analysis, carbamidomethyl (C) modification was fixed whereas oxidation modification (M) was not. One missed cleavage was permitted, and peptide charges were set at 2+ and 3+. Individual ion scores of more than 15 (p<0.05) were considered reliable and were subsequently used.

Functional annotations of OMV proteins were performed using the “clusters of orthologous groups” (COGs) functional classification (http://www.ncbi.nih.gov/COG) [51]. The subcellular localization of identified proteins was predicted using PSORTb version 3.0 (http://www.psort.org) [52].

### Fish, and immunization with OMVs or FKC

Naïve olive flounders (with an approximate weight of 55 g) were purchased from a commercial fish farm in Korea and acclimatized for 2 weeks at 21°C in aerated seawater. Formalin-killed *E. tarda* cells (FKC) were prepared by addition of 1% (v/v) formalin to an ED45 culture, followed by adjustment to an OD_600_ value of 1.0 using PBS. OMVs were prepared as described above and used after confirmation that no colonies formed when OMVs were plated onto TSA-2. Aliquots (0.1 ml) of FKC (1.2×10^8^ CFU/ml, 344 µg), or 10 µg amounts of OMVs, were intraperitoneally injected into olive flounder. The administred dosage was determined based on previous studies on immunization with OMVs originating from several gram negative bacteria [25], [27], [53], [54]. To evaluate expression of immune response-related molecules in fish, the kidneys of immunized olive flounder were dissected 0, 3, 7, and 12 hours post-infection (hpi), and 1.5 days post-infection (dpi), and stored in “RNA-later” solution (Ambion, Austin, TX) until all samples were available (N = 4).

All experimental protocols were approved by the Institutional Animal Care and Use Committee (IACUC) at Gyeongsang National University, Jinju, Republic of Korea (Approval Number: GNU-LA-17).

### Quantitative real-time PCR

Total RNA was isolated from kidney samples using the TRIzol reagent (Invitrogen, Scotland, UK) according to the manufacturer's protocol. After purification of 1 µg aliquots of mRNA using deoxyribonuclease I (Invitrogen), cDNAs were synthesized with the assistance of a high-capacity cDNA reverse transcriptase kit (Applied Biosystems, Foster city, CA) according to the manufacturer's protocol. cDNA samples were diluted 1∶4 (v/v) into nuclease-free water prior to amplification using quantitative real-time PCR (QRT-PCR). Primers were designed employing the Primer Express software of Real-Time PCR version 3.0 (Applied Biosystems). Primer names, sequences, and GenBank accession numbers are listed in Table 2. QRT-PCR was achieved using StepOne-Plus Real-Time PCR (Applied Biosystems) with a Fast-Start Universal SYBR Green Master Mix (Roche, Indianapolis, IN), according to the manufacturers' instructions. The amplification steps consisted of initiation at 95°C for 10 min, 40 cycles of denaturation at 95°C for 15 sec each followed by annealing at 60°C for 1 min, with next a melting curve analysis step featuring 1 cycle at 95°C for 15 sec, a hold at 60°C for 1 min, and a further hold at 95°C for 15 sec, to confirm that only a single amplicon was present. All reactions were performed in duplicate using olive flounder β-actin mRNA as an internal control. The relative standard curve method was applied to calculate standard errors of the mean (SEM). In statistical analysis, Student's *t*-test was used to determine differences between tests using FKC and OMV; p<0.05 was viewed as significant.

**Table 2:** Oligonucleotide sequences used in the present study.

Gene	Accession number	Primer name	Sequences (5′→3′)
β-actin	AU050773	WCUP090-F	CTGCCTTCACCTCCAAGAAG
		WCUP091-R	CTCCATGTCATCCCAGTTGGT
IL-1β	AB070835	WCUP279-F	ATGGAATCCAAGATGGAATGC
		WCUP280-R	TTAACTCTGATGATGGATGTT
IL-6	DQ884914	WCUP281-F	CCTGCACACCTACATGGTTCT
		WCUP282-R	TTGGGCATCTCTCTTTTACGA
TNFα	AB040448	WCUP294-F	GTCCTGGCGTTTTCTTGGTA
		WCUP295-R	CGTCCTCCTGACTCTTCTGG
IFNγ	AB435093	WCUP284-F	TGCAAGGATGAACAAAACCA
		WCUP058-R	AGAACTCGCCTCCTCGTACA
TLR22	AB109394	WCUP784-F	CTTGGCTTTGCTCTTTGACACA
		WCUP785-R	GGGCAGCAGCTCTCTCACA
TLR2	AB109393	WCUP781-F	CCACCGTCAGCGTCATAGAGA
		WCUP780-R	TTTTTCCCACCTGCCTTCAC

### Fish vaccination and challenge

Before vaccination, 10 naïve olive flounders in each group were intraperitoneally injected with ED45 (1.2×10^4^–10^9^ CFU/ml) and mortality was recorded over the next 28 days. A group of fish injected with 1.2×10^4^ CFU/ml of ED45 showed 70% mortality, and this dose was thus chosen as the challenge (data not shown). Twenty fish were vaccinated with 0.1 ml amounts of FKC (1.2×10^8^ CFU/ml; 344 µg) or 10 µg amounts of OMV, whereas fish injected with PBS served as controls. After acclimatization for 28 days, fish were challenged with 100 µl amounts of ED45 (1.1×10^4^ CFU/ml) by intraperitoneal injection, and mortality was recorded over the following 31 days. Vaccine efficacy was calculated as relative percentage survival (RPS = [1 minus vaccine group mortality/control group mortality]×100) [55]. In statistical analysis, Fisher's exact test was applied to compare survival differences between vaccinated and control groups at the p<0.0001 level.
